# Supplementary material for: Getting More Out of Biomedical Documents with GATE's Full Lifecycle Open Source Text Analytics
Source: PLoS Comput Biol. 2013 Feb 7;9(2):e1002854. doi: 10.1371/journal.pcbi.1002854 (PMC3567135; doi:10.1371/journal.pcbi.1002854)
Supplement: Dataset S1 — GATE software. Dataset S1 bundled with this paper contains a distribution of GATE (or see http://gate.ac.uk/download/). (TGZ) [file pcbi.1002854.s001.tgz › plos-gate/doc/codestyle.html]

GATE code style


GATE Code Style

``A computer language is not just a way of getting a computer to perform
operations but rather that it is a novel formal medium for expressing ideas
about methodology. Thus, programs must be written for people to read, and
only incidentally for machines to execute.''
*The Structure and Interpretation of Computer Programs*,
H. Abelson, G. Sussman and J. Sussman, 1985.


## Contents

This page describes the Java and HTML coding styles for GATE (and the
structure of the source and Web server trees). **Note** that this style
applies to the GATE codebase; you don't need to use this style for external
resources if you don't want to (but it would probably be a good idea if you
did).

- Code Conventions for the Java Programming Language (from Sun)- Example Java code- Example HTML- Notes on the structure of the source tree and the Web
        servers


## Java code style

Every bit of Java code in GATE should look like the following example.
(Note that "like" does NOT mean "sort of kind of fairly similar".)

Notes:

- Break lines before 80 characters.- Use 2-space indents in Java code, and follow the same style as the existing
    codebase.- Put a CVS ID flag in each file. Start each file with the standard header.- Make a javadoc comment on every class member, inc. private members.- Where appropriate (almost always!)
          use the Java collections library for collections.

**Debugging policy:**
if you want to debug a class using print statements and so on,
add this member:

```
  /** Are we debugging? */
  private static final boolean DEBUG = false;
```

then do stuff like

```
  if(DEBUG) {
    Out.pr("your debug stuff");
  }
```

Remember to set the variable false before you check in.

**Code example** (read this in a fixed-width font).

```
/*
 *  DataStoreRegister.java
 *
 *  Copyright (c) 1995-2010, The University of Sheffield. See the file
 *  COPYRIGHT.txt in the software or at http://gate.ac.uk/gate/COPYRIGHT.txt
 *
 *  This file is part of GATE (see http://gate.ac.uk/), and is free
 *  software, licenced under the GNU Library General Public License,
 *  Version 2, June 1991 (in the distribution as file licence.html,
 *  and also available at http://gate.ac.uk/gate/licence.html).
 *
 *  Hamish Cunningham, 23/Jan/2001
 *
 *  $Id: codestyle.html 12006 2009-12-01 17:24:28Z thomas_heitz $
 */

package gate;

import java.util.*;
import java.net.*;

import gate.util.*;
import gate.persist.*;
import gate.event.*;

/** 
 * Records all the open DataStores.
 */
public class DataStoreRegister extends HashSet {

  /** 
   * All the DataStore classes available. This is a map of class name
   * to descriptive text.
   */
  public static Map getDataStoreClassNames() {
    Map names = new HashMap();

    // no plugability here at present.... at some future point there should
    // be a capability to add new data store classes via creole.xml metadata
    // and resource jars
    names.put(
      "gate.persist.SerialDataStore",
      "SerialDataStore: file-based storage using Java serialisation"
    );

    return names;
  } // getDataStoreClassNames()

  /**
   * Adds the specified element to this set if it is not already
   * present. Overriden here for event registration code.
   */
  public boolean add(Object o) {
    return super.add(o);
  } // add

  /**
   * Removes the given element from this set if it is present.
   * Overriden here for event registration code.
   */
  public boolean remove(Object o) {
    boolean res = super.remove(o);
    if(res) fireDatastoreClosed(
      new CreoleEvent((DataStore)o, CreoleEvent.DATASTORE_CLOSED)
    );
    return res;
  } // remove

  /**
   * Removes all of the elements from this set.
   * Overriden here for event registration code.
   */
  public void clear() {
    Set datastores = new HashSet(this);
    super.clear();

    Iterator iter = datastores.iterator();
    while(iter.hasNext()) {
      fireDatastoreClosed(
        new CreoleEvent((DataStore) iter.next(), CreoleEvent.DATASTORE_CLOSED)
      );
    } // while
  }   // clear()


  ......


} // class DataStoreRegister
```


## HTML code style

Notes:

- Break lines before 80 characters.- Use 2-space indents. Indent tables so they're meaningful.- Put markup on separate lines to make things clearer (e.g. in lists).- If you use complex HTML you must: explain it in comments; make sure it runs on
        recent Netscape and IE; think about the implications for people who e.g. have
        vision problems and may not be able to get a speech generator to read your page;
        think about how long the page will take to go down a modem link.- Put a CVS ID flag in each file. Start each file with a header that includes
          meaningful title.- Always use size tags for images, so that the browser can display the rest of
            the page while waiting for the image to load. (You can use
            this script
            to generate size tags.)

(Read this in a fixed-width font).

```
<P>
GATE is a domain-specific software architecure and development environment
that supports:

<UL>
<LI>
researchers in Natural Language Processing and Computational Linguistics
<LI>
developers who are producing and delivering
<A HREF=http://www.dcs.shef.ac.uk/~hamish/thesis/le.html>Language
Engineering</A> systems.
</UL>

<P>
The GATE distrubution includes a free
<A HREF="http://www.dcs.shef.ac.uk/nlp/extraction/">Information
Extraction</A> system, which is free for all research purposes.

<P>
<TABLE BORDER=1>
  <TR>
    <TD>
    <B>Introduction</B>
    <BR> 
    </TD>
    <TD>
    <A HREF=http://www.dcs.shef.ac.uk/~hamish/GateIntro.html>A
    non-technical introduction</A>
    </TD>
    <TD>
    <A HREF=architecture.html>Some more technical details</A>
    </TD>
    <TD>
    <A HREF=publications.html>Links to publications</A>
    </TD>
  </TR>
  <TR>
    <TD>
    <B>Download</B>
    <BR> 
    </TD>
    <TD>
    To download, you first need to
    <A HREF=registration/reg-form.html>register here</A>,
    </TD>
    <TD>
    and a password will be emailed to you;
    </TD>
    <TD>
    then go to <A HREF=software.html>the download page</A>.
    </TD>
  </TR>
  <TR>
    <TD>
    <B>Documentation</B>
    <BR> 
    </TD>
    <TD>
    <A HREF=system_docs/gate_docs.html>System manuals</A>
    </TD>
    <TD>
    <A HREF=news.html>Known bugs</A>
    </TD>
    <TD>
    <A HREF=tipster.html>The TIPSTER architecture</A>
    </TD>
  </TR>
  <TR>
    <TD>
    <B>Misc</B>
    <BR> 
    </TD>
    <TD>
    <A HREF=users.html>Users of GATE</A>
    </TD>
    <TD>
    <A HREF=people.html>The people involved</A>
    </TD>
    <TD>
    <A HREF="http://www.dcs.shef.ac.uk/nlp/gate/contrib/michael.dorna/">A
    tutorial in German</A>
    </TD>
  </TR>
</TABLE>
```


## Notes on the structure of the source tree and web servers

The tree looks like this:

```
gate/build			home of makefiles and scripts, and gate.jar

gate/classes			.class, resource and .tcl files

gate/doc			documentation
gate/doc/internal		stuff we don't release, e.g. design notes
gate/doc/javadoc		autogenerated javadoc stuff
gate/doc/javadoc/internal	javadoc stuff including private members

gate/lib			.jars, .zips and associated licences

gate/misc			misc stuff like example jape grammars

gate/src			the source code

gate/src/gate/resources	resources (NOT CREOLE resources, 
				Java resources!) which get copied into the
				classes directory (and gate.jar)
gate/src/gate/resources/gate.ac.uk
				resources that get copied to the web servers
```

Web servers:

- http://gate.ac.uk/
  is the production server- http://redmires/
    is the linux development server, with lots of Java and tool
    documentation

Derwent and gate.ac.uk both serve files used by the test suite, and also any CREOLE metadata or GATE config data that we may want to distribute
centrally. These files are also available in the resources tree. Some
important files are:

```
creole/creole.xml		a central CREOLE metadata file
tests/creole.xml		a file for TestCreole tests
tests/gate.xml			a file for TestConfig tests
```

These are copied onto the web servers and live in:

```
gate/src/gate/resources/gate.ac.uk/creole/creole.xml
gate/src/gate/resources/gate.ac.uk/tests/creole.xml
gate/src/gate/resources/gate.ac.uk/tests/gate.xml
```

Not on the web servers, but in gate/classes and gate.jar:

```
gate/resources/creole/creole.xml
				CREOLE metadata file for builtins
gate/resources/gate.xml		central configuration data
```

Which live in:

```
gate/src/gate/resources/creole/creole.xml
gate/src/gate/resources/gate.xml
```

The resources tree, the test suite and the web servers all interconnect,
so that a) we can maintain the server files under CVS and b) we can have the
test files in gate/classes and in gate.jar
so we can run tests even when there is no web server accessible. The CVS
version of the tests and central CREOLE metadata is all kept in the resources
tree. Those under gate/src/gate/resources/gate.ac.uk are copied into
locations on derwent by the nightly build script.
